# Functional Status in Behavioral Variant Frontotemporal Dementia: A Systematic Review

**DOI:** 10.1155/2013/837120

**Published:** 2013-11-07

**Authors:** Thais Bento Lima-Silva, Valéria Santoro Bahia, Ricardo Nitrini, Mônica Sanches Yassuda

**Affiliations:** ^1^Neurology Department, University of São Paulo School of Medicine, Brazil; ^2^Gerontology Department, School of Arts, Sciences and Humanities of the University of São Paulo, Avenida Dr. Eneas de Carvalho Aguiar 255, 05403-900 São Paulo, SP, Brazil

## Abstract

The aim was to conduct a systematic review of studies that described the functional profile of patients with behavioral variant frontotemporal dementia (bvFTD), published between 2000 and 2013. The bibliographic search was conducted using the terms “frontotemporal dementia” and “frontotemporal lobar degeneration” in combination with “independence,” “functionality,” “basic activities of daily living,” “disability,” and scales that measure functional performance: “Disability Assessment for Dementia-DAD,” “Functional Activities Questionnaire (FAQ),” “Direct Assessment of Functional Status (DAFS).” To be included in the review, the study had to mention the characterization of the functional status of patients with bvFTD in the objectives of the study, using a previously validated instrument of functional assessment. Fourteen studies met this criterion. The reviewed studies suggested that individuals with bvFTD have greater functional impairment when compared to those with other subtypes of frontotemporal lobar degeneration or Alzheimer's disease. The studies documented a significant association between cognitive impairment and measures of functionality in these patients. The cognitive profile of patients may predict faster functional decline.

## 1. Introduction

bvFTD is a clinical syndrome characterized by progressive impairment in behavior and personality as well as social, cognitive, and functional abilities, which predominantly affects middle-aged adults [1, 2]. These changes stem from frontotemporal lobar degeneration (FTLD) secondary to a spectrum of heterogeneous pathologies [3, 4]. Despite recent advances in characterizing bvFTD, diagnosing this syndrome remains challenging. Patients may be erroneously considered to be cognitively unimpaired, while others are diagnosed with psychiatric disorders or Alzheimer's disease (AD) [5]. 

In 2011, a set of revised diagnostic criteria was proposed for the bvFTD based on recent publications and a consortium of international research groups [6]. The revised criteria were validated through the retrospective review of clinical records and the comparison of the accuracy of original and revised criteria in a sample of patients from 16 brain banks with autopsy-confirmed FTLD. With the revised criteria, a diagnosis of “possible” bvFTD requires three of the six clinically discriminated characteristics (loss of inhibition, apathy/inertia, loss of empathy, perseveration/compulsive behaviors, hyperorality, and dysexecutive neuropsychological profile). “Probable” bvFTD requires the additional features of functional disability and characteristic neuroimaging, whereas bvFTD “with definitive frontotemporal lobar degeneration” requires histopathological conformation or confirmation of pathogenic mutation. The new criteria are expected to contribute by allowing earlier diagnosis of the syndrome and differential diagnosis with psychiatric disorders. 

There is a consensus among researchers of bvFTD that, in the absence of specific biomarkers, investigating functional status is essential for the diagnosis and treatment of the syndrome, given that the impact on the activities of daily living can be used as a diagnostic parameter [6]. Yet, studies investigating the functional performance of patients with FTLD (bvFTD and other subtypes such as nonfluent progressive aphasia-NFPA and semantic dementia-SD) are scarce [2]. 

New studies characterizing the functional profile of patients with bvFTD are warranted as they can contribute to the diagnostic process and rehabilitation of patients. Moreover, functional dependence in bvFTD creates burden on caregivers and family members and generates additional social costs. Functional rehabilitation and compensation may alleviate some of these problems. Therefore, the aim of the present investigation was to carry out a systematic review of studies describing the functional status of patients diagnosed with bvFTD, published between 2000 and 2013.

## 2. Methods

This review is based on a literature search conducted in September 2013. The bibliographic search was carried out in Medline, PsycInfo, PubMed, LILAC's, SciELO, and ScienceDirect databases using the search term “frontotemporal dementia” and “frontotemporal lobar degeneration” in combination with “functionality”, “independence”, “basic activities of daily living”, “disability”, and names of scales that measure functional performance: “Disability Assessment for Dementia-DAD”, “Functional Activities Questionnaire (FAQ)”, and “Direct Assessment of Functional Status (DAFS)”, for retrieval of studies published between 2000 and 2013.

A total of 119 studies were retrieved, including articles, theses, dissertations, and book chapters. For inclusion in the review, studies had to cite in the objectives the characterization of the functional status of patients with bvFTD, using a previously validated functional assessment instrument. A total of 14 clinical studies met this criterion.

## 3. Results 

This review identified a limited number of studies which addressed the issue of functionality in patients with variants of FTLD using validated instruments. Among these studies, a small number included in their sample a significant number of patients with bvFTD. We found two research studies that addressed the correlation between functional performance and executive dysfunction in patients with bvFTD, when compared to other dementia cases [7, 8]. Two studies investigated functional performance compared to overall cognitive performance in patients with different dementia subtypes [9, 10]. Five studies compared functional performance among patients with various subtypes of FTLD [11–15] one study linked functional performance to emotional aspects [16], another correlated functional decline in bvFTD patients with caregiver burden [17], and finally, some studies compared the performance of patients with dementia in direct versus indirect assessments of functionality [8, 18, 19]. Two studies investigated rate of functional change [2, 10].

The reviewed studies suggested that individuals with bvFTD have greater functional impairment when compared to those with other subtypes of frontotemporal lobar degeneration or Alzheimer's disease. The studies documented a significant association between cognitive impairment and measures of functionality in these patients. In addition, it has been suggested that the cognitive profile of patients may predict faster functional decline. The selected studies are listed in Table 1, with their respective objectives, samples, cognitive and functional measures, and results. 

## 4. Discussion

The aim of the present study was to conduct a systematic review of studies investigating the functional profile of patients with bvFTD. A total of 14 clinical studies were identified whose aims were predominantly to compare the cognitive and functional performances of patients with bvFTD against other dementia types; provide a description of the functional profile of FTLD subtypes, comparing them with each other and noting which types presented greatest functional and cognitive decline; investigate the predictors of functional decline in bvFTD; compare indirect and direct functional performance of patients for different dementia subtypes; and assess scales for staging and measuring neuropsychiatric, dysexecutive, and functional symptoms in bvFTD. 

The findings of the studies included in this review are in line with earlier reports indicating that patients with bvFTD are younger than patients with other dementias given that early onset and rapid progression are frequent, particularly with regard to the bvFTD subtype [10]. Among the studies reviewed (see Table 1), patient age ranged from 50 to 60 years.

*Relations between Functional Impairment and Cognition*. Some studies included in this review suggested a significant association between cognitive and functional performance in patients with bvFTD. Razani et al. [8], for instance, reported that impaired verbal fluency was a predictor of functional incapacity, assessed directly by the DAFS-R and that performance on the WCST was moderately correlated with IADLs as reported by caregivers. Piquard et al. [7] found that worse performance on the MMSE was associated with functional incapacity, and that higher score on the CDR was a predictor of functional decline in patients with bvFTD. Additionally, Josephs et al. [2] reported that poor performance on executive, visuospatial, and language functions were indicative of more rapid progression in functional activities. These findings point to a relationship between cognitive and functional performance among patients with bvFTD that is not restricted to changes in executive functions. In other words, decline in other functions also appears to contribute to the functional deficits in this condition. In disagreement with the results outlined above, Mioshi et al. [9] failed to find any significant association between performance on the ACE-R and the DAD.

*Studies Comparing Functional Impairment across Dementia Types*. The studies reviewed suggested that individuals with bvFTD present greater functional impairment compared to patients with other FTLD subtypes or AD. Using the DAD, Mioshi et al. [9] showed that patients with bvFTD had poorer functional performance than patients with SD, NFPA or AD. This group had lower scores even on BADLs such as getting dressed, feeding, and hygiene. For IADLs, the worst performances were seen in finances, correspondence, and leaving the home. Poorer performances were also evident for the use of the telephone, domestic and leisure time activities, managing medications, and preparing meals, besides the impairments observed in AD patients. The authors highlighted the devastating impact of functional changes on the everyday routine of patients with bvFTD and the burden placed on their caregivers. These results were confirmed in later studies by the same group [15, 16].

Mioshi and Hodges [15] aimed to examine changes in activities of daily living (ADL), including subcomponents of the DAD and level of functional impairment in the FRS. The patients were subdivided into bvFTD pathological and phenocopy subgroups (patients who met diagnostic criteria but the disease did not progress), SD, and PNFA. The results indicated that pathological bvFTD, SD and PNFA groups showed significant decline in ADLs after 12 months, while the phenocopy subgroup did not. According to the FRS, the variants showed different levels of functional impairment. Virtually, all patients with bvFTD fell within the moderate, severe, very severe, or profound categories, whereas for the PNFA and SD groups, 25% fell within the mild category and there were no patients regarded as very severe or profound. The proportion of patients with PNFA in the severe category was about the double in comparison to patients with SD. In general, results suggested patients with bvFTD were more dependent on BADLs and IADLs than the other variants. In addition, functional performance and cognitive scores were significantly correlated and FTD variants showed differential annual rates of functional decline, with faster decline among bvFTD patients. 

The study by Wicklund et al. [12] examined the functional profile of patients diagnosed with AD, bvFTD, and primary progressive aphasia (PPA). Results showed that functional ability was moderately impaired in AD and bvFTD and mildly impaired in PPA. Self-care activities were the least impaired in all groups, whereas more complex ADLs, such as shopping and management of finances, were impaired early on. Communication ability was the least impaired, next to self-care for bvFTD and AD and the most impaired for PPA patients. 

Two studies failed to find differences among patients diagnosed with FTLD and AD on assessments of functionality. Carvalho et al. [13] found no significant difference in functional communication between AD and FTLD patients. Similarly, Bahia et al. [14] found no significant differences between patients with AD and FTLD on DAD scores. In their sample, DAD did not prove accurate for differential diagnosis of these dementia types. These negative results may be ascribed to the small sample size or to the fact that sample could not be stratified into FTLD subtypes.

Two of the reviewed studies examined functional performance of FTLD patients with direct assessment, involving the observation of the patient during functional activities. Razani et al. [17] found significant correlations between poor performance on the DAFS and increased burden and psychological distress of caregivers. Impaired performance on orientation, communication, finances and transport were associated with greater burden and hostility in caregivers. Comparing direct and indirect assessments of functionality in a mixed cohort of dementia patients, only one of which had FTLD, Bressan et al. [18] noted discordance between the opinion of caregivers/family members and actual observed performance of the patient, whereby informants often underestimated patients' potential to perform ADLs.

The reviewed studies highlighted the importance of carrying out functional assessment of patients with suspected bvFTD given the relevance of these changes for the diagnosis and clinical management of this dementia subtype. Some authors, such as Razani et al. [8], highlighted the dysexecutive profile of these patients and the significant impact on performing ADLs. Patients in the cited study exhibited difficulties starting tasks, estimating time, switching between tasks, and dealing with tasks of differing priorities. Also, subjects had problems controlling their impulses and exhibited deficits in chronological ordering, impatience, and emotional lability, directly impacting the performance of ADL. It is widely accepted that cognitive changes explain the functional deficits seen in patients with bvFTD. However, authors such as Mioshi et al. [15] suggested that apathy and impulsiveness, typical among this patient group, may also influence the functional deficits observed. 

Notably, some studies suggested that patients with bvFTD had lower survival rate compared to patients with AD, possibly associated with greater functional impairment [10]. Studies involving scales of severity and staging of bvFTD, such as the investigations by Marra et al. [11], Mioshi et al. [10], and Josephs et al. [2], support the hypothesis that the severity of bvFTD, together with its cognitive and neuropsychiatric symptoms, may modulate functional performance. 

The present work has limitations including the paucity of studies identified investigating the functional profile of patients with bvFTD. Additionally, some of the reviewed studies included a very small number of patients diagnosed with bvFTD, and this fact may render them more informative about dementia in general than bvFTD. It is noteworthy that the vast majority of studies in the current literature have investigated the functional profile of AD patients, healthy controls, and elderly with mild cognitive impairment [19–24]; therefore, the functional status of patients with FTLD requires further investigation. Future clinical and epidemiologic studies on FTLD should include instruments assessing functional performance in order to better characterize the patient profile. Such studies should have an impact on rehabilitation strategies geared towards patients with FTLD. Such findings should be relevant for the planning and delivery of support for families and may reinforce the need for tailored caregiver support that should take into account the disease stage and the functional limitations of the patient. 

## Figures and Tables

**Table 1:** Summary of clinical studies examining the functional profile of bvFTD patients

Author/year	Objectives	Sample	Cognitive/functionality measures	Results
Piquard et al., 2004 [7]	Study relationship between functional impairment and performance in planning activities in FTLD and AD	*N* = 40 patients (11 FTLD and 29 AD)	Dementia rating scale (DRS) Mini-mental state examination (MMSE) Cognitive difficulties scale (CDS) Physical self-maintenance scale (ADL) Instrumental activities of daily living (IADL) Social activities scale (SADL) Tower of London Dysexecutive questionnaire (DEX), the key search, the zoo map, and the six elements from the behavioural assessment of the dysexecutive syndrome (BADS)	No significant differences between bvFTD and AD on ADLs. No relationship between planning and executive function tests with functionality in either patient group An association was found between CDR and MMSE and functional performance in the two groups

Marra et al., 2007 [11]	Compare performance of demented elderly with different levels of severity in questionnaires on basic activities of daily living (BADL) and instrumental activities of daily living (IADL). Determine correlation between the ADL questionnaires applied	*N* = 90 patients (AD = 74, vascular = 7, mixed = 4, frontotemporal = 2, and others* = 3)	Clinical dementia rating Lawton-Brody index Katz index Functional activities questionnaire (PFAQ)	IADLs were more impaired at early stages of dementia and BADLs at more advanced stages

Mioshi et al., 2007 [9]	Assess ADLs and cognitive performance in subtypes of frontotemporal dementia, bvFTD, nonfluent progressive aphasia (NFPA), semantic dementia (SD) Ascertain the relationship between functional deficits and cognitive dysfunction of patients and compare against patients with AD	*N* = 59 patients with dementia (bvFTD = 15, NFPA = 10, SD 15, and AD = 19)	Disability Assessment for dementia (DAD) Addenbrooke's cognitive examination-revised (ACE-R) Clinical dementia rating scale (CDR)	For functional performance, bvFTD patients were most impaired (56% with functionality preserved), whereas NFPA and SD patients were less impaired (83% and 85% were functionally preserved). AD subjects had intermediate performance (76% showed preserved functionality). On ACE-R, the NFPA and SD groups had worse performance than bvFTD and AD patients. DAD performance did not correlate with cognitive measures, CDR, or time diagnosed with the disease

Razani et al., 2007a [8]	Correlate performance on executive function tasks with functional performance	*N* = 33 patients with dementia (3 FTLD, 21 AD, and 10 VD) and 35 healthy controls	Wisconsin card sorting test (WCST) Verbal fluency (FAS) Modified version of the Lawton-Brody measure (IADL) Direct assessment of functional status (DAFS)	Significant correlation between verbal fluency and DAFS. Moderate correlation between WCST and performance on subjective functionality scale

Razani et al., 2007b [17]	Assess the relationship between performance on ADLs in patients with mild dementia and the relationship with emotional burden of caregivers	*N* = 34 patients with dementia (5 FTLD, 23 AD, 5 VD, and 1 with undiagnosed form of dementia)	Direct assessment of functional status (DAFS) Lawton-Brody measure (IADL) Caregiver burden inventory (CBI) Brief symptom inventory (BSI)	Significant correlations between DAFS, caregiver burden (CBI), and psychological problems (BSI) On DAFS, impaired orientation, communication, finances, and transport were associated with greater burden and hostility in caregivers Impaired financial ability was strongest predictor of time dependent on caregivers, whereas impairment on transport domain predicted time since clinical diagnosis

Wicklund et al., 2007 [12]	Compare functional status in FTLD and AD	*N* = 100 with AD, 57 with bvFTD, and 61 with PPA	Instrumental activities of daily living (IADL)	Functional ability was moderately impaired in AD and FTD and mildly impaired in PPA For all groups, more complex ADLs were impaired early on, with relative preservation of self-care activities. The communication score was the least impaired next to self-care for FTD and AD and the most impaired for PPA patients

Bressan et al., 2007 [18]	Compare information from caregiver against direct assessment of patient functional performance	*N* = 25 patients with dementia, comprising 1 FTLD, 10 AD, 5 VD, 3 alcoholic dementia, 3 D. with Lewy Bodies, 1 corticobasal, and 2 dementias due to severe hypoxia	Pfeffer functional activities questionnaire (PFAQ) Observation of patients during simulated activities included in the PFAQ	Significant differences found between caregiver responses and direct observation of patient performance. Caregivers underestimated functional capacity of patients

Carvalho et al., 2008 [13]	Compare functional communication abilities in FTLD and AD patients.	*N* = 6 patients with AD and 8 with FTLD	Functional assessment of communication skills (ASHA-FACS)	Functional communication abilities were similar for patients with AD and FTLD. FTLD patients had worse performance on inference comprehension while AD patients had worse performance on basic transactions with money

Bahia et al., 2008 [14]	Verify utility of a functional and behavioral inventory in differential diagnosis between FTLD and AD	*N* = 12 patients with AD and 12 with FTLD	Frontal behavioral inventory (FBI) Disability assessment for dementia (DAD)	FIB proved accurate for differential diagnosis between FTLD and AD DAD failed to differentiate groups but helped assess severity of the condition

Mioshi and Hodges, 2009a [15]	Examine changes in activities of daily living (ADL), including subcomponents of initiation, planning, or execution of DAD	*N* = 10 patients with bvFTDphen, 6 with bvFTD path, 11 with SemDem, and 9 PNFA	Addenbrooke's cognitive examination-revised (ACE-R) Disability assessment for dementia (DAD)	Pathological bvFTD, SemDem, and PNFA groups showed significant decline in ADLs after 12 months, while the phenocopy subgroup did not Each variant showed different profiles of decline in subcomponents of ADLs

Mioshi et al., 2009b [19]	Describe activities of daily living in behavioral variant frontotemporal dementia, and correlate in caregiver and performance-based assessments	*N* = 18 patients with bvFDT	Addenbrooke's cognitive examination-revised (ACE-R) Disability assessment for dementia (DAD) Frontal systems behavior scale (frontal dysfunction) Assessment of motor and process skills	A model combining the global cognition and frontal dysfunction explained the variance on ADL performance. A qualitative rating distinguished between pathologic and phenocopy patients better than the performance-based assessment

Kipps et al., 2009 [16]	Compare emotion recognition behavior and social functioning in AD and bvFTD patients	*N* = 28, 14 patients with bvFTD and 14 with AD	Emotion hexagon Disability assessment for dementia (DAD) The Cambridge behavioural inventory (CBI)	bvFTD group had worse functional deficit, higher scores on the CBI, and poorer emotion recognition compared with the AD group Recognition of emotions was not correlated with DAD but instead with apathy on the CBI

Mioshi et al., 2010 [10]	Develop a novel tool to characterize FTLD severity based on functional disability and behavioral changes. Assess rate of change over time in 3 FTLD variants (bvFTD, SD, and NFPA)	*N* = 95 patients, 28 bvFTD, 21 NFPA, 26 SD, and 20 controls	Addenbrooke's cognitive examination-revised (ACE-R) Clinical dementia rating (CDR) Frontotemporal dementia rating scale (FRS)	FTLD progression differed among variants bvFTD patients progressed rapidly whereas subjects with SD presented slower evolution FDRS proved a good scale for staging and determining FTLD progression

Josephs et al., 2011 [2]	Relate behavioral, neuropsychological, and sociodemographic factors to determine predictive factors for functional decline in bvFTD	*N* = 97 patients with bvFTD	Clinical dementia rating (CDR) Trail making parts A and B Boston naming test Category fluency (sum of animals, vegetables, and fruits) Mini-mental status examination (MMSE) Neuropsychiatric inventory (NPI) Wechsler adults intelligence scale (WAIS-R) Magnetic resonance imaging analysis generating an anatomical subtype	Predominantly frontotemporal and frontal atrophy, worse performance on executive, visuospatial and language functions, loss of inhibition, agitation/aggression and nighttime behaviors, and greater age at disease onset were associated with faster rate of functional decline

Notes. FTLD: frontotemporal lobar degeneration, bvFTD: behavioral variant frontotemporal dementia, NFPA: nonfluent progressive aphasia, SD: semantic dementia, VD: vascular dementia, and AD: dementia of the Alzheimer type.

*Dementias caused by traumatic brain injury or by normal pressure hydrocephalus.
